# Propofol anesthesia improves stroke outcomes over isoflurane anesthesia—a longitudinal multiparametric MRI study in a rodent model of transient middle cerebral artery occlusion

**DOI:** 10.3389/fneur.2024.1332791

**Published:** 2024-02-13

**Authors:** Bart A. A. Franx, Geralda A. F. van Tilborg, Annette van der Toorn, Caroline L. van Heijningen, Diederik W. J. Dippel, Irene C. van der Schaaf, Rick M. Dijkhuizen, on behalf of the CONTRAST consortium

**Affiliations:** ^1^Translational Neuroimaging Group, Center for Image Sciences, University Medical Center Utrecht and Utrecht University, Utrecht, Netherlands; ^2^Department of Neurology, Erasmus University Medical Center, Rotterdam, Netherlands; ^3^Department of Radiology, University Medical Center Utrecht, Utrecht, Netherlands

**Keywords:** experimental stroke, acute ischemic stroke, reperfusion, general anesthesia, isoflurane, propofol, MRI, behavior

## Abstract

General anesthesia is routinely used in endovascular thrombectomy procedures, for which volatile gas and/or intravenous propofol are recommended. Emerging evidence suggests propofol may have superior effects on disability and/or mortality rates, but a mode-of-action underlying these class-specific effects remains unknown. Here, a moderate isoflurane or propofol dosage on experimental stroke outcomes was retrospectively compared using serial multiparametric MRI and behavioral testing. Adult male rats (*N* = 26) were subjected to 90-min filament-induced transient middle cerebral artery occlusion. Diffusion-, T_2_- and perfusion-weighted MRI was performed during occlusion, 0.5 h after recanalization, and four days into the subacute phase. Sequels of ischemic damage—blood–brain barrier integrity, cerebrovascular reactivity and sensorimotor functioning—were assessed after four days. While size and severity of ischemia was comparable between groups during occlusion, isoflurane anesthesia was associated with larger lesion sizes and worsened sensorimotor functioning at follow-up. MRI markers indicated that cytotoxic edema persisted locally in the isoflurane group early after recanalization, coinciding with burgeoning vasogenic edema. At follow-up, sequels of ischemia were further aggravated in the post-ischemic lesion, manifesting as increased blood–brain barrier leakage, cerebrovascular paralysis and cerebral hyperperfusion. These findings shed new light on how isoflurane, and possibly similar volatile agents, associate with persisting injurious processes after recanalization that contribute to suboptimal treatment outcome.

## 1 Introduction

General anesthesia (GA) is often used in the context of acute ischemic stroke treatment, both in the clinical and laboratory setting. For clinical use, GA can be induced during endovascular thrombectomy (EVT) to ensure patient immobility, pain management and airway protection (1). To that end, two GA types have been recommended: halogenated volatile anesthetics (e.g., iso-, des-, or sevoflurane), intravenous (i.v.) hypnotics (propofol), which may be used in combination (2). For preclinical stroke research, the same or similar GA types can be applied. GA is necessary because it ensures animal wellbeing during surgical procedures and monitoring (e.g., *in vivo* imaging), enabling the use of stroke models to study pathological and therapeutic pathways. The pharmacologic actions of GA are known confounders in assessment of stroke outcome, as there is ample evidence for GA-induced neuroprotection in preclinical models (3). Yet clinically, evidence is mixed, with multiple randomized clinical trials of GA versus conscious sedation conducted or still ongoing. In a meta-analysis of three randomized controlled trials (RCTs) and nineteen observational studies, as well as in a meta-analysis by the HERMES collaboration including seven RCTs on stent retriever efficacy, GA-assisted stroke treatment was associated with higher rates of disability and mortality compared with the non-GA treatment groups (4, 5). In another meta-analysis of seven single-center RCTs that randomized EVT patients to GA or non-GA treatment arms, it was found that GA improved recanalization rates and functional outcome (6). With several RCT results still on the horizon, the question whether GA ought to be applied during EVT is not settled yet.

Regardless of whether GA is detrimental or not, cases may present themselves where the stroke intervention team has no choice but to induce GA with the (combination of) drug classes recommended above. There is no consensus whether a certain GA class should be preferred, but several recent single-center retrospective studies comparing volatile- and propofol GA during EVT have suggested beneficial effects of propofol on functional outcome (7) or possibly mortality (8). This information is pertinent as it could further improve the outlook for ischemic stroke patients: EVT outcome may be differentially affected by GA type, but it also calls for a better understanding how different classes of anesthetics differ in the level of neurophysiological effects they confer.

From the experimental stroke field, there have been accounts on protective effects of propofol GA over volatile GA (mostly isoflurane) (9), however little is known about tissue fate beyond the acute phase, since most experiments were finalized within 24 h for *post-mortem* assays (9). Also, a potential mode-of-action explaining effects of isoflurane and propofol GA on experimental stroke outcome has not yet been elucidated. *In vivo* MRI enables serial assessment of lesion progression and hemodynamics from acute to chronic stages, which contributes to the understanding of stroke-related disease mechanisms and treatment effects (10). To that aim, the effects of anesthetic maintenance by isoflurane or propofol on post-stroke outcome measures were retrospectively studied in rats subjected to 90-min transient middle cerebral artery occlusion (tMCAO). MRI-based imaging markers of brain injury during, 0.5 h after, and four days after tMCAO were compared, while also assessing behavioral outcome on the fourth day.

## 2 Methods

Data presented here involve retrospective analyses of a subgroup of male rats used for pathomechanistic tMCAO studies (11), experiments from the propofol group are yet unpublished. Animal procedures were conducted according to guidelines of the European Communities Council Directive and approved by the Animal Experiments Committee of the University Medical Center Utrecht and Utrecht University.

### 2.1 Outcomes

The main experimental contrast of this study was isoflurane (inhalational) vs. propofol (i.v.) throughout experimental stroke induction and imaging sessions (see Sections 2.3 and 2.4 for details). Primary outcome for this experiment was lesion volume at day 4. The secondary outcome was behavioral outcome, quantified using a sensorimotor deficit score (SDS) before the follow-up MRI at day 4. Several secondary imaging markers of injury were sampled from the ischemic area that ultimately proceeded to infarction, i.e., the lesion core (see Section 2.10). These included the severity of cytotoxic and vasogenic edema, and cerebral hemodynamic aberrations, which were assessed throughout each imaging session. BBB permeability and cerebrovascular reactivity (CVR), imaging markers of tissue function, were also quantified in the lesion core during the final MRI session on day 4. BBB permeability was assessed on day 4 only, as (1) the present experimental design was confined by the design of the previous study from which these data are derived, and (2) cavitation starts to set in as the infarction progresses, the consequences of which invalidate the Patlak model of indicator tissue-uptake (as used in Section 2.8) at later stages of the injury.

### 2.2 Animals

Male adult *Sprague–Dawley* rats (*N* = 26, 11–13 weeks) from Charles-River were used, of which nineteen were included in the final analysis. Animals were housed with a littermate in a standard cage by a 12-h day/night cycle, with *ad libitum* access to food and water. Rats were habituated for one week before experiments. MRI experiments were conducted during middle cerebral artery occlusion, 0.5 h after recanalization and at follow-up after four days. Animals were included if an ischemic lesion was detected in the right striatum or neocortex, with an ischemic volume larger than 30 μL during MCAO. This led to the exclusion of seven animals. Due to the retrospective nature of this study, animals could not be randomized to anesthesia condition. At the time of experimental conduct, researchers could not be blinded to GA type due to the delivery method of anesthesia (inhalation vs. injection). However, data processing and analysis pipelines—such as for lesion segmentation—were automated and required minimal human input, mitigating investigator bias.

### 2.3 Animal handling

Anesthesia was maintained throughout the microsurgically-induced tMCAO (see Section 2.4) and imaging experiments, either with 2% isoflurane or i.v. propofol (40 mg∙kg^−1^∙h^−1^; details below). As no reasonable method exists to translate dosage between humans and rodents, dosages were chosen in alignment with the literature on experimental stroke research (12) and functional hemodynamic studies in rats (13, 14). Expired CO_2_ was continuously monitored with a capnograph (Microcap, Oridion Medical 1987 Ltd., Israel) to guide ventilation volume, breathing rate and anesthesia depth. Body temperature was maintained at 37 ± 0.5°C with a feedback-controlled heating pad. Blood oxygen saturation and heart rate were continuously monitored by pulse-oxymetry throughout MRI sessions. Animals received ophthalmic cream (Duratears^™^ Z, Alcon, Switzerland) before surgery and MRI, and were subcutaneously injected with 0.9% NaCl (1 mL/100 g) postoperatively and after MRI sessions to replenish lost fluids. Body weight was monitored daily before and after surgery. Predefined conditions for humane endpoints were not exceeded and there was no mortality before the end of the experiment. Additional 0.9% NaCl injections were administered if deemed necessary by visual inspection of hydration status (1 mL/100 g, subcutaneous).

### 2.4 Experimental workflow

Three days before tMCAO, baseline behavioral tests were conducted. Before tMCAO and MRI, rats were anesthetized with 4% isoflurane in air:O_2_ (4:1) for endotracheal intubation, followed by mechanical ventilation with 2% isoflurane in air:O_2_ (4:1). Animals received ophthalmic cream (Duratears^™^ Z, Alcon, Switzerland) and a 10 mg/kg subcutaneous injection of lidocaine (Xylocaine 5%, AstraZeneca, Sweden) in the throat area prior to tMCAO. After these preparations, which typically lasted 15 min, rats were switched to their assigned anesthetic maintenance agent, i.e., 2% isoflurane (IsoFlo®) or propofol (Fresenius Kabi, The Netherlands) (40 mg∙kg^−1^∙h^−1^).

Transient middle cerebral artery occlusion was induced as described by Zea Longa et al. (15). Briefly, the common carotid artery (CCA), internal carotid artery (ICA) and external carotid artery (ECA) were exposed and dissected. The CCA was temporarily ligated while the ECA was cauterized and arteriotomized. The ECA was reflected along the ICA so a silicon-tipped filament (4–0, Doccol Corporation, USA) could be advanced 22 mm until resistance was felt. The incision was temporarily closed using Tergaderm (3 M, United States) and, without discontinuation of anesthesia, the animal was transported to the MRI scanner for the first examination. After 90 min of MCAO, the filament was removed and a 2 mg/kg intra-incisional injection of bupivacaine (Levobupivacaine 0.25%, Fresenius Kabi, Germany) was administered before the wound was sutured. The animal was transported back to the MRI system for a second examination, after which it was allowed to recover on a heating mat. After four days, behavioral assessment was repeated. Lastly, animals were re-anesthetized using 4% isoflurane and switched to the appropriate maintenance agent and dosage for the third and final MRI examination.

### 2.5 Behavioral testing

An adapted battery of behavioral tests was performed several days before stroke induction (pre-measurement) and four days after stroke, before the final MRI session, to calculate an overall sensorimotor deficit score (SDS) (16) as previously described (11). Briefly, animals were scored consecutively on spontaneous exploratory mobility and gait disturbance, lateral resistance (when pushed sideways), whisker-guided forelimb placing, forelimb grasping- and strength on a horizontal bar, and finally postural signs when being held by the base of the tail. Total SDS ranged from 0 (no deficit) to 22 (maximum deficit). The pre-measurement was not further analyzed because no functional deficits were found before MCAO.

### 2.6 MRI

MRI experiments were conducted on a horizontal bore 9.4 T MR system (Varian, Palo Alto, CA, United States), equipped with a 20.5-cm gradient coil able to generate 400 mT/m. A Helmholtz volume coil (Ø 80 mm) and inductively coupled surface coil (Ø 25 mm) were used for signal transmission and detection, respectively. Anesthetized rats were placed in a MR-compatible stereotactic holder and restrained with a headset and tooth bar and mechanically ventilated (see Section 2.3).

Each imaging session included diffusion-weighted and T_2_-weighted imaging. Dynamic susceptibility contrast-enhanced MRI (DSC-MRI) was executed with an intravenous bolus injection of Gd-DTPA (Gadobutrol, Bayer Healthcare, Berlin, Germany) (0.35 mmol/kg, i.v.). During the third MRI examination on day 4, the status of the BBB was assessed by serial quantitative T_1_ mapping after the Gd-DTPA bolus injection for DSC-MRI. Six DSC-PWI experiments and three T_1_-mapping experiments were excluded due to Gd-DTPA injection failure and technical issues. To characterize CVR, the blood oxygenation level-dependent (BOLD) MRI signal was quantified during a hypercapnic vascular challenge induced by 0.1 L∙min^−1^ CO_2_, producing a gas mixture containing 9% CO_2_. The challenge constituted of a three-minute baseline, a subsequent four-minute exposure to the gas stimulus (starting as the capnograph indicated more than 0.5% end-tidal CO_2_). Details on pulse sequences are shown in Supplementary Table S1.

### 2.7 Image processing

All images were processed using FSL 5.0 (17). Images were corrected for inhomogeneity (18) before brain extraction, performed with FSL BET (19). Image coregistration was performed with FSL FLIRT (20). When registering images from the subacute phase (post-stroke day 4), non-linear registration was additionally applied with the FSL Non-linear Image Registration Tool to mitigate effects of space-occupying edema (causing midline shift). The unweighted (b = 0) image from the diffusion-weighted MRI scan acquired during MCAO served as the internal reference (hereafter: native space), to which each subsequent b0 image from different time points was registered.

Mean apparent diffusion coefficient (ADC) maps were obtained by averaging three ADC maps acquired with diffusion-sensitive gradients applied along the cardinal directions x, y or z. Quantitative T_1_ and T_2_ maps were calculated by derivative-based full least-squares fitting of complex-valued data (21). Maps of cerebral blood flow (CBF) were calculated by circular deconvolution of tissue concentration curves using an arterial reference curve obtained from the contralateral hemisphere (22). CBV was calculated by numeric integration of the tissue concentration curve, truncated at the 400^th^ image to minimize contrast agent recirculation effects. Mean transit time (MTT) was the quotient of CBV and CBF.

### 2.8 Blood–brain barrier permeability

R_1_-maps were used for estimation of the blood-to-brain indicator transfer constant (*K_i_*) of the Gd-DTPA contrast agent across the BBB, indicative of leakage, using a generalized Patlak plot approach (23), where the reference concentration was obtained from a tissue region where there is reversible communication with the blood, for which a large ROI over the muscle adjacent to the skull was selected. Next, the following was plotted:


$$X\left(t\right)=\frac{\int_{0}^{t}C_{RT}\left(\tau \right)d\tau }{C_{RT}\left(t\right)}$$

on the abscissa, and


$$Y\left(t\right)=\frac{C\left(t\right)}{C_{RT}\left(t\right)}$$

on the ordinate axis, where *C* denotes the concentration in the tissue and *C_RT_* the concentration in the reference tissue (i.e., muscle). Voxelwise *K_i_* was then calculated by regressing *X(t)* against *Y(t)* and taking the slope of the fitted line.

### 2.9 Cerebrovascular reactivity

To reduce the influence of intra-individual variation on BOLD response quantification, signal magnitude was normalized (voxelwise) to baseline values before the hypercapnic challenge. The BOLD signal from the contralateral hemisphere was selected using a mask and time-averaged. From the resulting one-dimensional contralateral signal, the time index of maximum change from baseline was selected, from which was assumed to be the relative time in the experiment where BOLD-signal change would have been maximal in the entire brain during the hypercapnic challenge. The CVR map was obtained by calculating mean BOLD response percent-change over 100 images centered on the time of maximal BOLD response, relative to mean BOLD signal at baseline.

### 2.10 Region-of-interest (ROI) analyses

To track lesion evolution, semi-automatic delineation of cytotoxic edema on ADC-maps during occlusion (ischemic area) and vasogenic edema on T_2_-maps at four days (infarct area) were performed as described previously (11). From these lesion masks the following ROI was derived (in native space): irreversible acute ischemic injury that proceeded to infarction, i.e., *lesion core* = final lesion ∩ acute ischemic area. Contralateral homologues were obtained by aligning mirrored copies of the lesion core ROI to the source image in native space. Next, the ipsi- and contralateral lesion core ROI was aligned with parametric maps (ADC-, T_2_-, CBF-, CBV-, MTT-, K_i_-, and CVR maps). Notably, perfusion estimates are prone to inadvertent within- or between-subject variability stemming from variable bolus injection speeds. To mitigate this effect, the mean value underlying the lesion core mask was calculated and always expressed as a percentage of the mean from the contralateral homologous area (a fair control), producing a relative regional perfusion index (rrCBF, rrCBV and rrMTT).

All lesion volumes were measured in absolute volumes (μL) in native space. When it was necessary to correct for inter-individual variability in brain size (i.e., to conduct an unpaired t-test of ischemic lesion volumes), these values were normalized by the volume of the ipsilesional hemisphere, yielding hemispheric lesion volumes.

### 2.11 Statistical analyses

Standardized effect sizes are reported in square bracket with ±95% confidence intervals. Assumptions such as the normality of residuals were checked where appropriate before parametric testing.

To test the effect of GA type on primary (final lesion volume) and secondary (SDS) outcomes, least-squares regression was performed using these outcomes as dependent variables in univariate analyses. For both these models, GA type was a fixed explanatory variable. The absolute volume and the location of the initial ischemic lesion (cortico-subcortical, subcortical, or diencephalic), measured during occlusion, were used as control variables. For the final lesion volume, ordinary least-squares assuming a Gaussian distribution of the response variable was performed, whereas for the SDS, a generalized linear model was fitted assuming the Poisson distribution for count data.

Additional secondary outcome parameters were analyzed using linear mixed models, which were applied to compare group averages where longitudinal or otherwise non-independent observations were concerned (see below). Subject (i.e., rat) was always set as random effect. Residual degrees-of-freedom were restricted by Kenward-Roger approximations. Bonferroni-corrections were always applied for post-hoc testing.

ADC- and T_2_-values from the lesion core were compared individually over time (occlusion vs. 0.5 h vs. day 4) and GA type (propofol vs. isoflurane), which were regarded fixed.

Relative regional perfusion indices (i.e., rrCBF, rrCBV and rrMTT) were compared individually over time (occlusion vs. 0.5 h vs. day 4) and GA type (propofol vs. isoflurane), which were regarded fixed.

Lastly, from the lesion core region-of-interest, BBB permeability (operationalized by the indicator transfer constant ‘K_i_’) and CVR were obtained and compared using Hemisphere (ipsilesional vs. contralesional) and GA type (propofol vs. isoflurane) as fixed effects.

Statistical analyses were performed in R version 4.0.3 using packages *tidyverse* (24), *car* (25), *lme4* (26), *emmeans* (27) and *stargazer* (28).

## 3 Results

For the isoflurane-anesthetized group, eighteen animals were used, of which seven animals were excluded due to insufficient ischemic lesion volume. For the propofol-anesthetized group, eight animals were used, and all were included in the final analysis. There were no significant differences in temperature and blood oxygenation between the two groups at any time.

**Figure 1:**
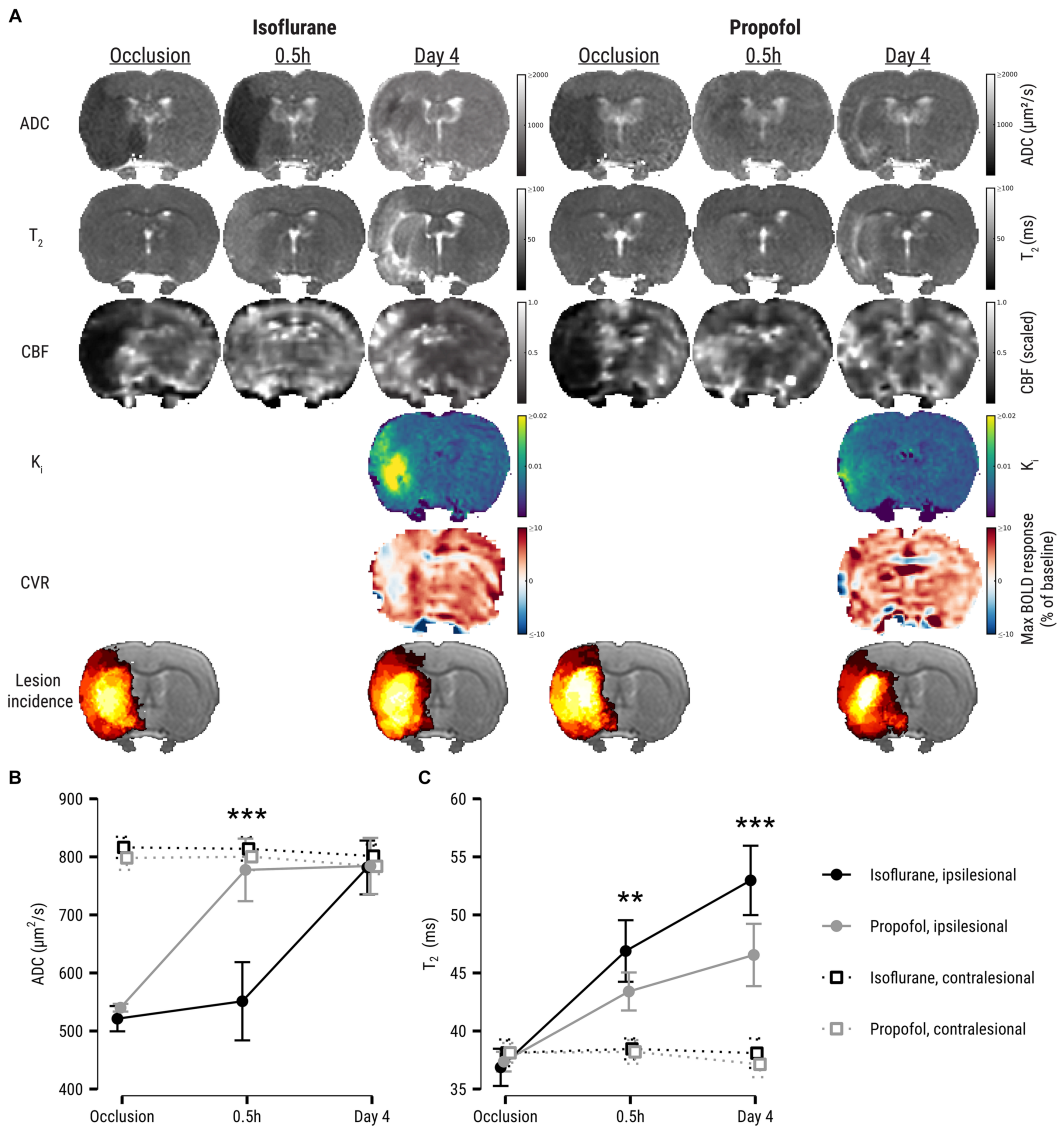
**(A)** ADC-, T_2_-, CBF-, K_i_-, and CVR maps at all experimental time points of two representative examples from the isoflurane and propofol groups. CBF maps are dimensionless and have been rescaled to 0–1 range to aid interpretation. The 6th row shows group-averaged lesion incidence maps (%) during occlusion (ADC-derived ischemic lesion) and at day 4 (T_2_-derived final infarct). It can be qualitatively appreciated from these incidence maps that the volumetric difference between initial ischemic lesion and final infarction is larger in the propofol group compared to the isoflurane group. Lesion evolution is depicted in **(B)** as apparent diffusion coefficient (ADC) and **(C)** T_2_ values (mean ± SD) in the ipsilesional core and contralesional homologue during occlusion and after recanalization. Asterisks denote significant differences within ipsilesional ROIs between anesthesia groups. ADC, apparent diffusion coefficient; CBF, cerebral blood flow; K_i_, blood-to-brain indicator transfer constant; CVR, cerebrovascular reactivity; BOLD, blood-oxygen-level-dependent. **p < 0.01; ***p < 0.001.

Figure 1A shows ADC-, T_2_-, CBF-, K_i_-, CVR maps from representative examples from the isoflurane and propofol groups, as well as group-specific lesion incidence maps. The hemispheric lesion volume of the ischemic area during occlusion was comparable between groups: 0.15 ± 0.10 in the isoflurane group and 0.17 ± 0.12 in the propofol group (*t* = −0.46, *p* = 0.65) (not shown).

A significant association between GA type and final lesion size at day 4 was detected after correction for ischemic lesion volume during filament occlusion: subjects that received isoflurane anesthesia had significantly larger final lesion volumes (132 vs. 55 μL); *p* = 0.004, [−3.0, −0.4]). See Table 1 for details. Also, GA type significantly affected the SDS at day 4, associating with higher functional deficiency scores after isoflurane GA (6.0 vs. 3.8, *p* = 0.01, [−4.2, −0.35]). See Table 1 for details.

**Table 1:** Results of least-squares regression of general anesthesia type and control variables against lesion volume or sensorimotor deficit score.

		*Dependent variable*	
		Final lesion volume	SDS
**Model coefficients (β ± 95% CI)**	GA type (isoflurane)	77.14^**^ (32.76, 121.51)	0.46^*^ (0.10, 0.83)
	Acute ischemic lesion volume	150.16^***^ (95.36, 204.96)	0.45^*^ (0.02, 0.89)
	Lesion type (subcortical)	−12.50 (−75.94, 50.94)	−0.13 (−0.69, 0.43)
	Lesion type (diencephalic)	−50.09 (−135.58, 35.40)	−2.00^**^ (−3.47, −0.53)
**Model performance**	F	19.98	
	Log Likelihood		−41.16
	RMSE	38.45	2.36
	Residual df	14	14
	Adjusted R^2^	0.81	
	Adjusted R^2^ (Nagelkerke)		0.90

GA, general anesthesia; RMSE, root mean square error; SDS, Sensorimotor deficit score. *p < 0.05; **p < 0.01; ***p < 0.001.

During occlusion, mean ADC in the lesion core was similar in the propofol and isoflurane groups (Figure 1B). Shortly after recanalization, ADC in the lesion core increased to a higher level in the propofol group compared to the isoflurane group (*p* < 0.0001, [−6.6, −3.7]). ADC values equalized in the subacute phase four days after stroke. Further, mean T_2_ in the ischemic lesion was lower in the propofol group early after recanalization (*p* = 0.002, [0.6, 2.8]), as well as after four days (*p* < 0.0001, [1.9, 4.3]) (Figure 1C).

**Figure 2:**
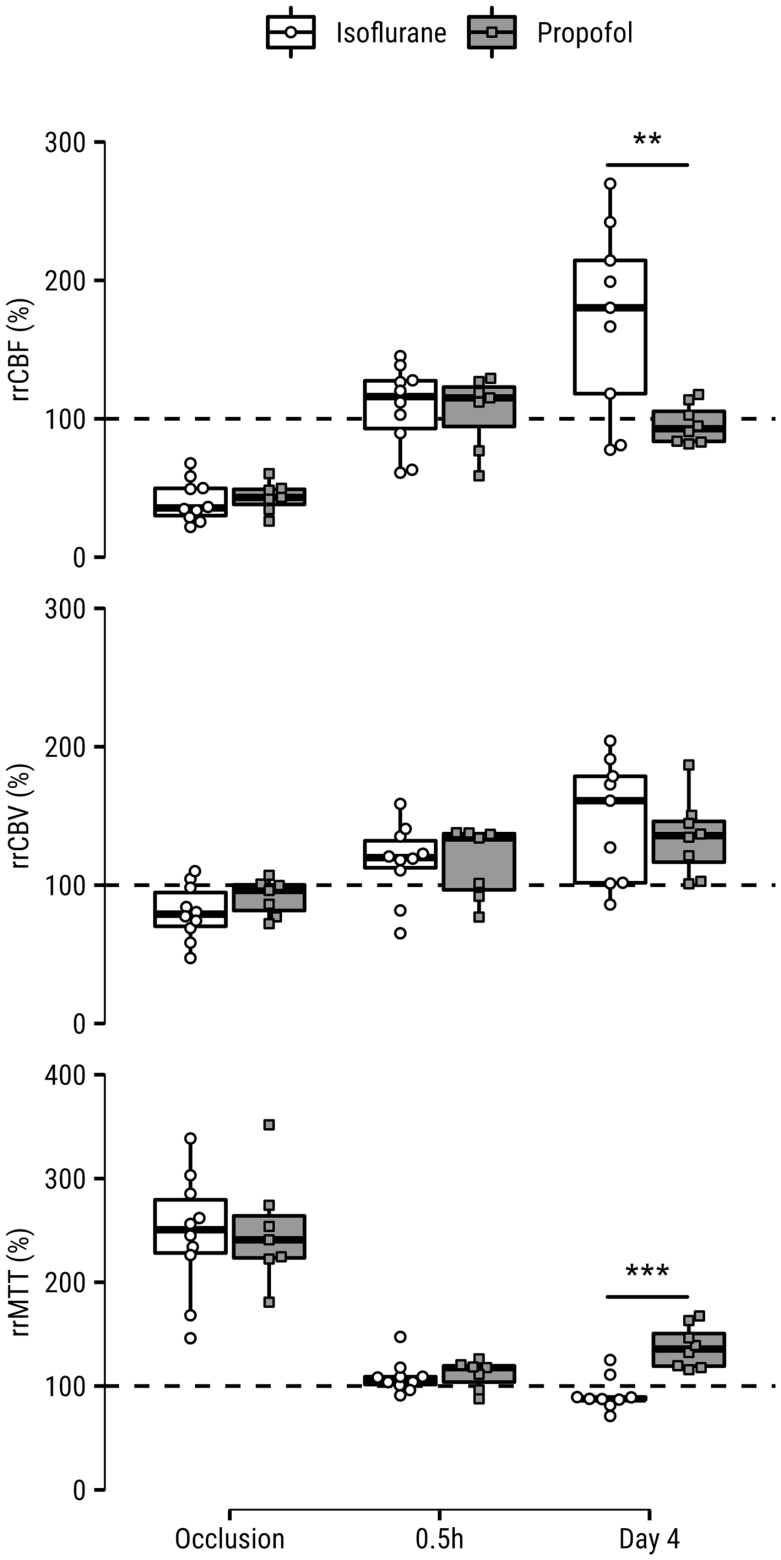
Relative regional cerebral blood flow (rrCBF), cerebral blood volume (rrCBV) and mean transit time (rrMTT) measured in the (post-)ischemic lesion core of rats under isoflurane or propofol anesthesia during occlusion, 0.5 h after recanalization and four days after recanalization. Values are expressed as a percentage of the contralesional mean from a homologous area. Dashed lines indicate hypothetical normal level of percent-difference per hemodynamic parameter. **p < 0.01; ***p < 0.001.

Relative regional perfusion values in the lesion core are shown in Figure 2. There were strong main effects of *Time* for all perfusion indices, but the values were similar for both GA groups during occlusion (all parameters similarly indicated hypoperfusion) and 0.5 h after recanalization (all parameters equally demonstrated restoration of perfusion). rrCBF was higher in the isoflurane group after four days (*p* = 0.008, [0.6, 3.2]), although a main effect of GA type was not detected. There was a main effect for GA type on rrMTT (*p* = 0.009): blood transit times were significantly shorter in the isoflurane compared to the propofol group four days after tMCAO (*p* = 0.0002, [−3.3, −1.1]).

**Figure 3:**
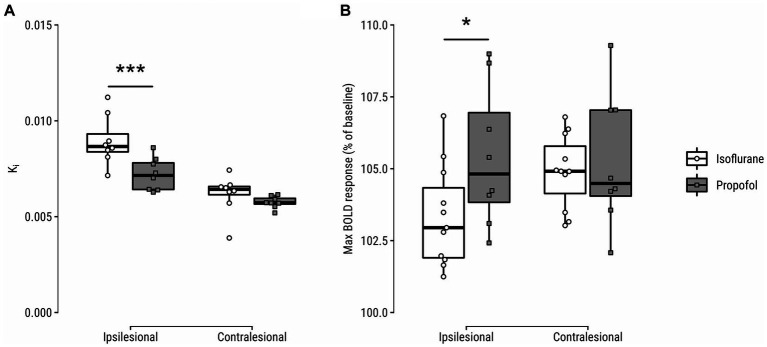
**(A)** Blood-to-brain transfer constant (K_i_) and **(B)** hypercapnic challenge-induced blood-oxygen-level-dependent (BOLD) response (*%-difference of peak blood-oxygenation-level-dependent (BOLD) signal from baseline during CO_2_ inhalation*) in the lesion core and contralateral homologue on day 4 after stroke. *p < 0.05; ***p < 0.001.

K_i_ was significantly lower in the infarct core at day 4 (*p* = 0.0009, [0.7, 3.1]) in propofol- vs. isoflurane-maintained animals (Figure 3A). CVR to a hypercapnic challenge was significantly larger in the infarct core of propofol-maintained animals at post-stroke day 4, as compared to isoflurane-maintained animals (*p* = 0.03, [−2.8, −0.1]) (Figure 3B).

## 4 Discussion

This study compared the effects of isoflurane and propofol anesthesia in a longitudinal imaging study of experimental acute ischemic stroke. The primary outcome, lesion size at follow-up after four days, was significantly reduced in the propofol group compared to the isoflurane group, even after correction for initial ischemic lesion size. Similarly, functional deficit scores were significantly reduced at follow-up after propofol GA compared to isoflurane GA. Earlier studies on effects of propofol in experimental stroke models have already shown benefit when propofol is administered shortly before or after recanalization (9), but imaging marker(s) for a possible mode-of-action for propofol have not been described thus far. Data presented here demonstrate that tissue ADC reduction, an imaging marker for cytotoxic edema (29, 30), was equal in both GA groups during MCAO but rapidly dissipates after recanalization in the lesion core of the propofol group, indicative of restoration of the energy-dependent cellular ion homeostasis (31). The lingering ADC reduction after recanalization in the isoflurane group indicates persisting metabolic failure (32), which could explain worsened outcomes of this group. Clinically, ADC normalization after recanalization has been associated with good outcome—although various trials confirm or disprove this relationship (33).

Propofol reduced post-stroke vasogenic edema within the post-ischemic area, indicated by lower T_2_ values as compared to the isoflurane group. This process develops secondary to BBB permeability, where intravascular fluids and proteins leak into the parenchyma, paving the way for (lethal) space-occupying brain edema (34). Correspondingly, BBB permeability, measured in the subacute phase, was reduced in the propofol group. Crucially, during and shortly after occlusion, no differences in relative hemodynamics were detected between study groups, suggesting that better outcomes in the propofol group were not associated with relative regional perfusion differences early in the experiment. Yet later in the subacute phase, rrCBF was significantly elevated in the isoflurane group, in line with our previous work (35), while appearing normal in the propofol group. Post-ischemic hyperperfusion beyond the acute phase likely associates with the severity of acute ischemic damage (36), which was less severe early after tMCAO under propofol GA, which may have dampened the effects of the subsequent infarction process. Several works demonstrated that a post-stroke CBF overshoot in the (sub)acute phase locally associates with BBB dysfunction (37) and reduced CVR (37, 38). The present analysis shows amelioration of both these imaging markers in the propofol group, evidencing improved vascular health in the post-ischemic lesion core. Importantly however, while relative CBF appeared normal after four days in the lesion territories of the propofol group, other parameters were still perturbed: CBV was still elevated like in the isoflurane group, resulting in prolonged MTT.

Propofol GA improved neurologic outcomes without notable differences with isoflurane on cytotoxic edema or relative regional perfusion in the lesion core during occlusion, which can generate new hypotheses about possible pharmacodynamic effects of propofol and isoflurane during ischemia. Volatile- and propofol GA both act through GABA-ergic inhibition (39, 40), decreasing CMRO_2_ (41), yet they cause distinct effects on cerebral hemodynamics (41, 42). Volatile gas GA induces a dose-dependent increase in CBF for isoflurane at levels above 1 MAC [approximately 1.12% isoflurane in adult rats (43)]. Comparative studies using equipotent dosages have shown that propofol reduces CBF as opposed to isoflurane in both rats (44) and humans (45, 46). Presumably, through preservation of the CBF-CMRO_2_ coupling mechanism (41), propofol may have been better able to protect the (post-)ischemic lesion compared to isoflurane, which decouples CBF from local oxygen demand (41). Thus, while *relative* perfusion indices were similar between the experimental groups, *absolute* values of cerebral perfusion during and shortly after MCAO—which cannot be readily quantified using DSC-MRI—may not have been. In line with this, GA class-dependent effects on CVR are also worthwhile to consider, as this mechanism contributes to steady-state vascular parameters such as CBF and CBV. In our study, CVR was measured by BOLD-MRI combined with a hypercapnic stimulus. Based on the similar contralesional BOLD response in the isoflurane and propofol groups, we conclude that CVR was similarly left intact under isoflurane and propofol anesthesia, at least using the present dosages. Future work comparing dose-dependent effects of volatile gas- and intravenous anesthesia on CVR could provide valuable information regarding which GA agent best preserves CVR, which is a relevant parameter in EVT procedures where GA is involved.

There are some limitations to this study. Due to its retrospective nature, randomization and blinding could not be performed, however imaging analyses were automated to reduce risk of investigator bias. Further, for a brief 15-min induction period, anesthesia was introduced with isoflurane in both groups, carrying minor risks of drug–drug interaction for the propofol group at the start of the experiment. This decision was grounded in practicality, relying on rapid elimination of isoflurane from the body. In humans, isoflurane is cleared from well-perfused organs such as the brain within 30 min (47). Since rodent metabolism is considerably faster, this duration represented the upper bound of our estimation of when the isoflurane left from induction would be eliminated. In addition, the experimental design employed here precluded continuous measurement of blood pressure, which would require invasive surgery (incompatible with behavioral testing) or intravascular implantation of telemetric devices (incompatible with MRI). Future studies may elect to sacrifice either of these experimental outcome parameters for the purpose of measuring blood pressure. Lastly, these experiments were performed on healthy adult male rodents, but an earlier meta-analysis of preclinical literature indicated that GA neuroprotection fails in female rodents or comorbid models (9), which might imply that present results are not translatable. However, the authors also emphasized that their finding is limited by a very small pool of studies that included only the protective effects of volatile gas anesthesia, generalization to other GA classes is therefore overhasty. Nevertheless, it stands to reason that a sample of adult males is not fully representative of the clinical stroke population, and further elucidation of class-dependent GA effects in a diverse rodent sample will be useful.

While (randomized) clinical trials and meta-analyses of GA vs. conscious sedation in stroke patients continue to provide crucial information about the effect of GA on EVT outcomes, information on which GA classes were employed (propofol, volatile gas, or otherwise) remains under-reported. In earlier studies, GA was unstandardized such that mixtures of GA classes were used for induction and/or maintenance, or GA agents were used in lower concentrations in control groups, weakening the intervention contrast, which hampers the ability to draw conclusions about agent-specific effects. Previous work has already called for more complete reporting and improved protocolization of GA regimes (48), the present data further emphasizes this need. Finally, our findings may promote further clinical investigations into the possible role of anesthesia protocols in enhancing therapeutic efficacy and patient enrollment in recanalization procedures, in line with recent STAIR recommendations (49).

In conclusion, the present preclinical study comparing isoflurane vs. propofol GA during and after tMCAO demonstrates ameliorating effects of propofol on experimental stroke outcomes. In comparison to isoflurane GA, propofol GA was associated with reduced final infarct size and functional deficit, diminished cytotoxic and vasogenic edema, reduced BBB disruption and preserved cerebrovascular reactivity. These results can generate new hypotheses about pharmacodynamic actions of isoflurane and propofol GA and may be useful in the search for improved GA management in human stroke victims.

## Data availability statement

The raw data supporting the conclusions of this article will be made available by the authors, without undue reservation.

## Ethics statement

The animal study was approved by Animal Experiments Committee of the University Medical Center Utrecht and Utrecht University. The study was conducted in accordance with the local legislation and institutional requirements.

## Author contributions

BF: Conceptualization, Data curation, Formal analysis, Investigation, Methodology, Software, Visualization, Writing – original draft, Writing – review & editing. GT: Data curation, Formal analysis, Methodology, Supervision, Writing – review & editing. AT: Data curation, Investigation, Methodology, Software, Writing – review & editing. CH: Investigation, Writing – review & editing. DD: Formal analysis, Writing – review & editing. IS: Formal analysis, Writing – review & editing. RD: Conceptualization, Funding acquisition, Methodology, Supervision, Writing – review & editing.
