# Supplementary material for: Propofol anesthesia improves stroke outcomes over isoflurane anesthesia—a longitudinal multiparametric MRI study in a rodent model of transient middle cerebral artery occlusion
Source: Front Neurol. 2024 Feb 13;15:1332791. doi: 10.3389/fneur.2024.1332791 (PMC10897009; doi:10.3389/fneur.2024.1332791)
Supplement: Supplementary file 1 [file Data_Sheet_1.PDF]

Supplementary Material

Supplementary Table 1: MRI pulse sequence data

	DWI	T_2w	DSC-MRI	Look-Locker	BOLD-MRI
**Sequence**	2D SE-EPI (8-shot)	2D SE-EPI (8-shot)	2D GE-EPI (single-shot)	2D GE-EPI (8-shot)	2D SE-EPI (single-shot)
**TR / [TE] (ms)**	2000 / [31]	3000 / [30, 50, 80, 190]	164 / [13]	5000 / [9]	705 / [4]
**TI (ms)**	20	20	15	[16 … 4616]<br><br>(24 equidistant echoes)	15
**FOV**	33.75 × 33.75 mm^2	33.75 × 33.75 mm^2	31.2 × 31.2 mm^2	33.6 × 33.6 mm^2	31.2 × 31.2 mm^2
**Data matrix size**	224 × 224<br><br>(25 slices)	224 × 224<br><br>(25 slices)	64 × 64<br><br>(6 slices)	128 × 128<br><br>(15 slices)	72 × 72<br><br>(13 slices)
**Voxel size (μm^3)**	150 × 150 × 600	150 × 150 × 600	487.5 × 487.5 × 1200	262.5 × 262.5 × 1200	433.3 × 433.3 × 1200
**Other**	b = 0, 1454 s/mm^2		0.6 mm slice gap		

Note: DWI = diffusion-weighted imaging. T_2w = T_2-weighted imaging. DSC-MRI = dynamic susceptibility contrast-enhanced MRI. BOLD = blood oxygenation level dependent. FOV = field of view.
